# Exploring reasons for discontinuing use of immediate post-partum intrauterine device in Nepal: a qualitative study

**DOI:** 10.1186/s12978-020-0892-5

**Published:** 2020-03-18

**Authors:** Mahesh C. Puri, Saugat Joshi, Aayush Khadka, Erin Pearson, Yasaswi Dhungel, Iqbal H. Shah

**Affiliations:** 1 Center for Research on Environment, Health and Population Activities (CREHPA), Kathmandu, Nepal; 2 Harvard T. H. Chan School of Public Health, Boston, USA; 3 Ipas, Chapel Hill, USA

**Keywords:** Discontinuation, Post-partum IUD, Nepal

## Abstract

**Background:**

Postpartum intrauterine device (PPIUD) use remains very low in Nepal despite high levels of unmet need for postpartum family planning and the national government’s efforts to promote its use. This study investigates reasons for continuing or discontinuing PPIUD use among Nepali women.

**Methods:**

We conducted in-depth interviews (IDIs) with 13 women who had discontinued PPIUD use and 12 women who were continuing to use the method 9 months or longer following the insertion. All interviews were audio recorded, transcribed, translated into English, and analyzed using a thematic approach.

**Results:**

Women discontinued PPIUD for several reasons: 1) side effects such as excessive bleeding during menstruation, nausea, back and abdominal pain; 2) poor quality of counselling and, relatedly, mismatched expectations in terms of device use; and 3) lack of family support from husbands and in-laws. In contrast, women who were continuing to use the method at the time of the study stated that they had not experienced side-effects, had received appropriate information during counselling sessions, and had the backing of their family members in terms of using PPIUD.

**Conclusion:**

Experiencing side-effects or complications following PPIUD insertion and poor quality of family planning counselling were the two main reasons for discontinuation. Family members appeared to play a major role in influencing a woman’s decision to continue or discontinue PPIUD suggesting that counseling may need to be expanded to them as well. Improving quality of counselling by providing complete and balanced information of family planning methods as well as ensuring sufficient time for counselling and extending PPIUD service availability at lower level clinics/health posts will potentially increase the uptake and continued use of postpartum family planning, including PPIUD, in Nepal.

## Plain English summary

The use of contraception following delivery (referred to as the postpartum period) remains low in Nepal. In particular, the use of postpartum intrauterine device (PPIUD) is very low despite its many advantages and the government’s continued efforts to promote its use to protect against unintended pregnancies. In addition, little is known about the continuation of PPIUD use or the reasons for stopping its use.

In this study, we examined reasons for discontinuation through in-depth interviews with 13 women who had discontinued PPIUD use and 12 women who were continuing to use the method 9 months or longer since insertion in one of six tertiary public hospitals in Nepal. We found side-effects, poor quality of counseling, mismatch between expectations and experiences, and lack of family support to be the major reasons for PPIUD discontinuation. In contrast, women who were continuing to use PPIUDs at the time of the study were more likely to report not having faced any health-related side-effects and having a supportive family.

These results emphasize the need to improve the quality of counseling provided to women in Nepal and to possibly expand the scope of counseling to include other family members.

## Background

Postpartum intrauterine contraceptive device inserted within 48 h of delivery (PPIUD) is a safe, effective, and efficient method of meeting women’s need for long-acting but reversible method of contraception [1–3]. PPIUD is a particularly attractive method of contraception in a country like Nepal where women face several barriers to easily and repeatedly access healthcare services. The Nepali government has recognized the potentially critical role of intrauterine devices (IUDs) in meeting the contraceptive needs of women and has been actively promoting it over the last decade. However, these efforts have had limited success with data from 2016 suggesting that IUD use among married women in the country was merely 1.4% [4].

Reasons for low uptake range from limited provision of IUDs to a lack of staff trained in providing family planning counseling and services to pre-existing biases against IUDs among both providers and the general public [5–7]. A recent intervention by the International Federation of Gynecology and Obstetrics (FIGO), in coordination with the Nepal Society of Obstetricians and Gynecologists (NESOG) and the Ministry of Health and Population, sought to address some of these challenges by training healthcare workers on providing family planning counseling during antenatal care visits and on integrating PPIUD services with delivery. Pradhan et al. (2019) have showed that this intervention had a small but significant effect on PPIUD uptake [8].

Ensuring greater use of PPIUD among women looking for long-acting, reversible methods of contraception does not only involve scaling-up PPIUD access but also reducing rates of discontinuation among women who have been using the device. Recent data from 2016 Nepal Demographic and Health Survey indicate that about one in four (28.2%) of IUD users discontinue its use within 12 months [4]. The literature on IUD discontinuation has identified several reasons for why women choose to discontinue, including desire to have another child, facing health-related complications, and changing preferences for contraceptive methods [9–13]. However, little is known about whether these reasons adequately capture the motivation of Nepali women who continue or discontinue PPIUD.

Understanding users’ experience and perspectives that contribute to their decision to continue or discontinue using PPIUD can help develop quality family planning counseling and service provision in Nepal. Given the high unmet need for postpartum family planning, especially for a long-acting reversible method such as PPIUD, and the priority of Nepal government is giving to addressing it, insights from users’ experiences and perspectives are critical to improve service provision.

## Data and methods

### Program description

Our study explores reasons for PPIUD discontinuation within the context of a training and information intervention conducted by FIGO in six tertiary-level hospitals in Nepal. The intervention comprised of training maternity care providers in postpartum family planning counseling, PPIUD insertion techniques and complication management. Hospitals were selected based on geographical location and high volume of deliveries (between 6000 and 11,000 a year) and were pair-randomized into two groups of three based on location and annual number of deliveries. Details about the program and the subsequent evaluation have been published elsewhere [8, 14].

### In-depth interviews

We conducted in-depth interviews with 25 women enrolled in the PPIUD evaluation study across all six hospitals in Nepal. All 25 women had PPIUD inserted as part of the FIGO intervention and were selected for interview at around 9 months following the date of PPIUD insertion. Women were selected using a two-step process: first, all six study hospitals prepared a list of women who were continuing to use PPIUD or had reportedly discontinued using the method by around 9 months following insertion. Second, the study team randomly sampled between 2 and 3 women per hospital among PPIUD continuers and discontinuers for in-depth interviews. The final sample included 13 women who had intentionally discontinued using PPIUD and 12 women who were continuing to use.

To conduct these interviews, we developed interview guides in English and then translated them to Nepali. We did not back-translate these guides although a researcher not involved with the initial translation thoroughly checked the guides for translation accuracy. Two trained researchers conducted all interviews between July 2017 and September 2017. Interviews took place at the woman’s residence in a private space without the presence of any other family member. Only one woman selected for the interview refused to participate.

### Interview protocol

Before each interview, researchers asked participants to review a consent form and encouraged them to ask questions regarding the interview or the PPIUD intervention evaluation study more broadly. Upon receiving written consent, we asked participants if they would consent to having the interview audio recorded. All participants who consented to the interview also consented to the audio recording. We conducted interviews in Nepali, Maithili, or Abadhi languages depending on the participant’s preference. On average, interviews lasted for about an hour (range: 46–80 min). A trained researcher transcribed and translated all interviews into English for the purposes of analysis.

### Analysis strategy

We used a thematic approach to analyze the interviews. As a first step, two researchers independently read the transcripts and developed an initial set of codes. Some of these codes were subsequently combined and modified to develop a more refined codebook. Then, a researcher not involved in the initial step reviewed and finalized the codebook. All interviews were then coded based on the finalized codebook and content codes were grouped into major thematic categories. We organized the text, coded, and grouped the data into relevant themes using the ATLAS.ti software (Version win 7.0, Scientific Software Development, GmbH, Berlin, Germany).

### Ethical approval

This study was approved by the Ethical Review Board of the Nepal Health Research Council.

## Results

### Participant profiles

All interviewed women were between the ages of 16 and 34 years and had between one and three living children. Almost all women reported being literate but only three had completed secondary school. Very few women reported being employed at the time of interview. Most women reported being first time users of any family planning method. Among those who had used any family planning method in the past, only one had used an IUD.

### Drivers of PPIUD discontinuation

Women who discontinued PPIUDs did so for three main reasons: health-related concerns and side-effects; poor quality of counseling received and, relatedly, mismatched between expectations and experience of using PPIUDs; and the influence of family members.

Eight out of the 13 women reported that they discontinued using the IUD because of side-effects they experienced while using the method. The most reported side-effects were feeling a pricking sensation, dizziness, back pain, and abdominal pain. Some women also reported experiencing heavy menstrual bleeding. A number of women also reported that the side-effects they experienced interfered with their day-to-day activities.

*“I had heavy bleeding after insertion which led to severe back pain and pricking sensation … I started fearing and as my child is small, I didn’t know what to do. Therefore, I decided to discontinue using.”*

- 17 years old, one living child, PPIUD discontinuer

*“The thread had come out also I couldn't breastfeed my baby because of pricking sensation. So, I went to cut the thread. I also felt dizziness around 18* ^*th*^*day since insertion and hence I got it removed”.*

*-* 21 years, one living child, PPIUD discontinuer

In contrast, women who were continuing to use PPIUD at the time of the interview were far less likely to report having experienced any side-effects or disturbances to their ability to execute their roles and responsibilities.

*“It has been 11 months since I have been using copper T (IUD), it has suited me. I also liked the fact that it prevents pregnancy for 12 years. There is no side-effect for me till now”.*

*-* 19 years old, one living child, PPIUD continuer.

Respondents also brought up the issue of poor-quality counseling as a reason for discontinuing PPIUD use. Women who had been adequately informed about the advantages and disadvantages of the PPIUD during counseling sessions were more likely to be using the method.

*“They had told me that if I use copper T, I will be safe for 12 years. It is very safe and effective method of family planning, you won’t get pregnant while using it. It is better to use copper T instead of using injection. … ... They also told me its other advantages and likely problem I may have. I liked whatever information I received”.*

*-* 22 years old, two living children, PPIUD continuer

Most women who discontinued using the PPIUD, however, reported that they had not received any guidance from their family planning counselor about the potential risks related to PPIUDs. They claimed that they were only told about the length of time the PPIUD would provide protection for but were not advised on substituting methods or issues surrounding incorrect placement of the device or potential side effects. Some women even claimed that they were not shown what an IUD looked like during the counseling sessions.

*“I didn’t receive enough information on Copper-T (IUD). There are chances that the copper-T (IUD) may not be in the right place. They didn’t talk in detail about such issues. They should have clearly informed me about it”.*

*-* 21 years old, one living child, PPIUD discontinuer.

*“They didn’t give me any information. They only told me about Copper-T (IUD) and suggested me to insert it for my benefit. I wasn’t told anything about other family planning methods”.*

*-* 21 years old, one living child, PPIUD discontinuer.

*“I hadn’t seen Copper-T (IUD) before and they also didn’t show me the method while giving me information about it”.*

*-* 19 years old, one living child, PPIUD discontinuer.

Women who discontinued PPIUD also cited mismatched expectation in terms of the device, which they attributed primarily to not being provided enough information on the method during counseling sessions.

*“It didn’t match my expectations. The thing is they didn’t give full information before insertion. After using copper-T (IUD), I started having problems like pain in my stomach, loss of appetite and had headaches. Even though this method works for 12 years, I removed it after 2-3 months of use. I didn’t like this method”.*

*-* 31 years old, one living child, PPIUD discontinuer

A third reason women cited for discontinuing PPIUD use was the influence of family members. Women cited a lack of empowerment to make choices regarding contraceptive use and (mis) information from family and community members as key reasons that influenced their decision to discontinue PPIUD use.

*“My family members and neighbors used to tell me to remove the Copper-T and also said that my breast milk is not coming out because of the use of the method. I personally didn’t want to remove it. But, my father-in-law and sister-in-law told almost everyone in the village that I have used PPIUD. Thereafter, even the villagers used to tell me to remove it. So, I had no option but to remove the method”.*

*-* 19 years old, one living child, PPIUD discontinuer

*“My husband agreed and allowed me to insert the Copper-T (IUD) in front of the nurses at the hospital. But, after returning home he listened to the negative comments from the villagers and started fighting with me for having inserted the Copper-T. My mother-in-law and sister-in-law also provided wrong information to my husband. After listening to them, my husband started arguing with me. Some [family members] said that I will have a cancer while some said it will be difficult to remove it later. So, I had to remove it”*

*-* 21 years old, one living child, PPIUD discontinuer

Women who were continuing to use the PPIUD were more likely to report having a family environment that enabled and supported them in terms of their contraceptive choices.

*“I received good support. My husband told me, copper T will be good so use it. My mother-in-law also told me, it will be good to use it, your baby is also small now. They had told me, you have two children now there are chances of getting pregnant again, now you have enough babies so you can use it”.*

*-* 22 years old, two living children, PPIUD continuer

### Users’ perspectives in improving PPIUD services

Respondents also provided multiple suggestions to improve PPIUD service provision in Nepal with an eye towards reducing discontinuation of its use. They emphasized the need to ensure timely and complete counseling, including the displaying of the actual IUD during counseling sessions. One woman said:

*“They should give the detail information about PPIUD and also about all the other available methods of family planning. I don’t know much about the methods of family planning. I only know some information about condom, Depo, Norplant and Copper-T. I haven’t seen any of these methods as well. They should show every method and inform women about it”.*

*-* 19 years old, one living child, PPIUD discontinuer

Since a number of women were also advised by their family members to remove the PPIUD, expanding counseling sessions to include family members would seem an effective strategy for the uptake and continues use of PPIUD. Moreover, women particularly stressed on providing clear and detailed information on PPIUD. As many women were unsure about the health risks in longer run, they suggested the providers to take adequate time and explain in detail about the advantages and disadvantages of the method. Along with that, they also suggested to give them choice to select a method, including PPIUD.

*“What I didn’t like is that they (providers) did the insertion in a rush. They just said about copper-T (PPIUD) and not about other methods. They just said that it would be better to insert copper-T at that time (delivery) as the uterus is open. I didn’t understand about the method properly. I think they should have taken more time to explain me the method and other alternative I have”.*

*-* 28 years old, two living children, PPIUD discontinuer

To facilitate easy and timely access, some women also suggested providing the PPIUD service from the nearby health post enabling them to go for a routine checkup in time. Apart from that, women also suggested the counseling to be provided from the health posts as well.

*“It is difficult for those whose house is far to go to hospital carrying baby. Also, it is difficult to go to hospital from places where there is no facility of transportation. So, it would be nice to have facility for checkup at health post in the village. Women need not go far place carrying babies. Many women don’t go for checkup as it is far away; if there is facility nearby then it would be easy for women to go for checkup if they have any problem. It would be good if there is a service for checkup at health post”.*

*-* 27 years old, one living child, PPIUD discontinuer

## Discussion

Most of the discontinuers initially considered PPIUD as a good method, but discontinued its use for several reasons. Commonly cited reasons for removal included family pressure against using PPIUD, side-effects such as excessive bleeding during menstruation, nausea, back and abdominal pain, and poor quality of counselling. For majority of the discontinuers, their experience was worse than what they had expected by using PPIUD. For example, many women expected that PPIUD will last longer without having any health complications or side-effects but they experienced health problems. The lack of family support specially from their husband, relatives and in-laws regarding side-effects and negative opinions or rumors on PPIUD like having tumor in stomach were also the reasons of discontinuation.

Several studies have documented similar findings on reasons for discontinuing PPIUD. One prospective observational study carried out at tertiary hospitals in India for 1 year reported that 245 women who discontinued PPIUD (out of 5622), 38.8% discontinued due to bleeding problems and 37.5% due to the pain in the abdomen. Other cited reasons such as personal decision, partial expulsion and fear of expulsion accounted for the remaining 24% of discontinuations [15]. Similarly, another study, also in India, concluded that, 54 of 2072 women who discontinued PPIUD, 19 discontinued due to menstrual abnormalities like excessive bleeding and 13 women discontinued due to pain in the abdomen [16].

Most of the women who continued PPIUD for 9 months or longer had completed secondary level education, had supportive family and experienced no complication regarding their health as compared to those who discontinued PPIUD. Furthermore, women who received adequate and timely counselling about the method during and after insertion and those who were aware about advantages of PPIUD were more likely to continue using it.

Post-partum family planning is critical to delaying the next pregnancy and short birth intervals that are associated with negative newborn, infant and child health outcomes. Long-acting reversible methods, especially PPIUD, is well suited in the context of Nepal where deliveries at health facilities are rising and some women reside in remote areas. For many women, this offers an opportunity for timely protection against an unintended pregnancy and saving time and cost for subsequent visit to health facility of family planning. Yet, postpartum family planning and especially the use of PPIUD remains low in Nepal. Promoting use of a less commonly used and known method, such as PPIUD, is a formidable challenge. For a less commonly used method, the experience is sporadic giving rise to rumors or one-sided, generally negative, reports. Therefore, stronger and well-designed efforts are required to provide complete, balanced, and high quality information and counselling including method description, their advantages and disadvantages. High quality counselling require sufficient time for women to make an informed choice. In the Nepalese context, involving husband and mother-in-law in information and counselling is critical for the uptake and continued use of PPIUD. In-depth interviews also highlight the need for PPIUD service to be available at health posts enabling timely and faster access.

## Conclusions

Findings suggest that most women who discontinued PPIUD experienced side-effects, received poor quality counselling, and received no or little information on PPIUD and other methods of family planning. Therefore, good quality and balanced counselling particularly on what is expected in terms of side effects and complications following PPIUD insertion would help women in making the appropriate choice and more likely to continue using the method or timely switch to another method.

Since family plays a major role and can be very influential in the Nepalese context, counselling should also involve women’s family members, particular husband and mothers-in-law. Strengthening the skills of providers for providing quality counselling and insertion services are critical to meeting women’s need for postpartum protection and fertility goals.

By comparing the perspectives and experiences of continuers and discontinuers, this study highlights the importance of providing complete and balanced information on all methods. The need to learn each woman’s particular situation, including her familial context, to tailor the counseling that is best suited for her needs and situation emerged as a significant new programmatic finding from this study.

## Data Availability

Once de-identified, the dataset used and/or analysed during the current study will be available from the corresponding author on reasonable request.

## Abbreviations

ANC: Antenatal care
CREHPA: Center for Research on Environment Health and Population Activities
FIGO: International Federation of Obstetricians and Gynecologists
FP: Family Planning
IDI: In-depth interviews
IUD: Intra-uterine device
NESOG: Nepal Society of Obstetricians and Gynecologists
PPIUD: Post-partum intra-uterine device
